# 1530. Reactivation of Latent HIV in CD4 T Cells Procured from Patients on ART by Autologous CD8 T cells or Fas Ligand

**DOI:** 10.1093/ofid/ofad500.1365

**Published:** 2023-11-27

**Authors:** Daniel Leshin-Carmel, Ziv Sevilya, Gabriel Brik, Orit Gal-Garber, Daniel Waiger, Klaris Riesenberg, David Hassin

**Affiliations:** Assuta Ashdod, Ashdod, HaDarom, Israel; Assuta Ashdod, Ashdod, HaDarom, Israel; Assuta Ashdod, Ashdod, HaDarom, Israel; Robert H. Smith Faculty of Agriculture, Food and Environment, the Hebrew University, Rehovot., Rehovot, HaMerkaz, Israel; Robert H. Smith Faculty of Agriculture, Food and Environment, the Hebrew University, Rehovot, HaMerkaz, Israel; Faculty of Health Sciences, Ben Gurion University of the Negev, Beer Sheva, HaDarom, Israel; Assuta Ashdod, Ashdod, HaDarom, Israel

## Abstract

**Background:**

HIV persists in a long-lived infected CD4 T cell reservoir, which harbors an integrated transcriptionally latent virus. We reported that cytotoxic CD8 T cells conjugate with, and kill, autologous HIV-infected CD4 T cells isolated from all stages of HIV infection including reservoir cells of patients under antiretroviral therapy (ART) (image 1). This killing is attenuated by HIV Nef protein. We also previously reported that FasL presented by the cytotoxic T cells interacts with Fas on the surface of the target cells, resulting in the apoptosis of the target cells. Wajant demonstrated that Fas/FasL interaction can result in the target cell activation through NFkB, whereas NFkB binding site is present in the Long Terminal Repeat of HIV. Therefore, we hypothesize that the interaction of FasL and autologous CD8 T cells with CD4 T cells can result in the reactivation of latent HIV.

Conjugation and apoptosis of CD4 T cells by autologous CD8 T cells procured from patients on ART and analyzed by ImageStream.

**Figure:**
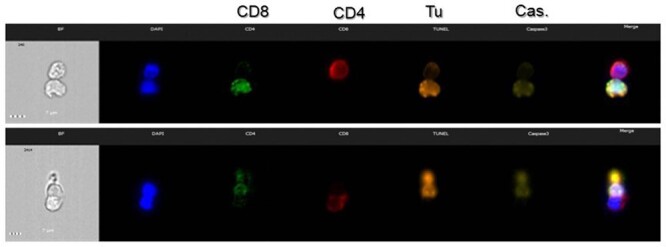

The CD4 T cells in apoptosis are positive for activated caspase 3 (yellow) and DNA fragmentation/ TUNEL assay (orange). CD4 T cells (green), CD8 T cells (red), DAPI (blue).

**Methods:**

CD4 T cells and CD8 T cells were procured from the PBMC of 20 HIV-infected patients on ART with undetectable viral load and 10 healthy volunteers. The cells were isolated using magnetic beads. Resting memory CD4 T cells (CD25^-^, CD69^-^ and HLA-DR^-^) were isolated using a two-step bead depletion purification procedure. These cells were then incubated with soluble FasL (sFasL) and autologous CD8 T cells, for 2 and 16 hours. P24 was looked for in the supernatant by ELISA, and the expression of viral proteins was looked for inside CD4 T cells using immunohistochemistry and confocal microscopy

**Results:**

CD4 T cells and resting memory CD4 T cells, procured from PBMC of HIV-infected patients on ART, showed HIV reactivation after incubation with sFasL and autologous CD8 T cells. This reactivation was demonstrated by the appearance of P24 in the supernatant (figure 1). HIV proteins p24, gp120, and Nef appeared inside the CD4 T cells (Image 2). HIV reactivation was not demonstrated in the CD4 T cells procured from HIV-infected patients that were incubated without sFasL or autologous CD8 T cells for 18 hours (figure 1).

Immunohistochemistry and confocal microscopy of CD4 T cells incubated with sFasL for 18 hours.

**Figure:**
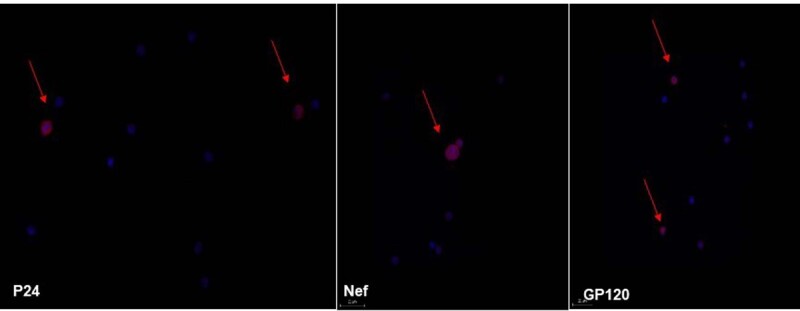

The cells were then incubated with a primary fluorescent antibody against HIV proteins (P24, Nef, GP120) and a secondary antibody ALEXA594 (red). The cells were also marked with DAPI (blue). We can see the expression of HIV proteins inside the CD4 T cells (red arrows).

ELISA for the expression of P24 in the supernatant of CD4 T cells procured from HIV-infected patients, following different manipulations

**Figure:**
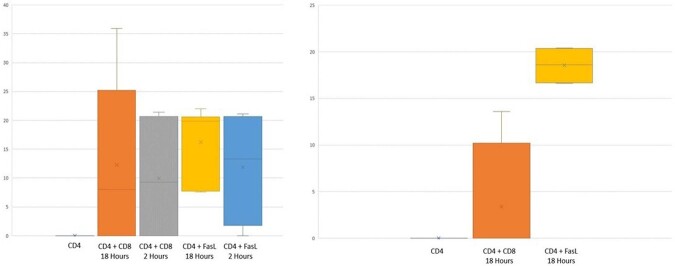

On the left, we note positive ELISA for P24 in the supernatant of the CD4 T cells of patients 11 to 16 (pg/ml). On the right, we note positive ELISA for P24 in the supernatant of the latent CD4 T cells of patients 17-20 (pg/ml).

**Conclusion:**

HIV manipulates the cellular immune system in two ways; First, HIV attenuates CD8 T cells killing of HIV-infected CD4 T cells by the HIV-Nef protein. Second, reactivation of latent HIV by CD8 T cells and sFasL, that results in further infection of additional CD4 T cells.

**Disclosures:**

**All Authors**: No reported disclosures

